# The relationships of sleep apnea, hypertension, and resistant hypertension on chronic kidney disease

**DOI:** 10.1097/MD.0000000000003859

**Published:** 2016-06-10

**Authors:** Chih-Ping Chang, Tsai-Chung Li, Liang-Wen Hang, Shinn-Jye Liang, Jen-Jyn Lin, Che-Yi Chou, Jeffrey J.P. Tsai, Po-Yen Ko, Chiz-Tzung Chang

**Affiliations:** a Division of Cardiology; b College of Medicine, China Medical University; c Department of Healthcare Administration, College of Health Science, Asia University; d Graduate institute of Biostatics, College of Public Health, China Medical University; e Sleep Center; f Division of Nephrology, China Medical University Hospital; g Department of Bioinformatics and Medical Engineering, Asia University, Taichung, Taiwan.

**Keywords:** apnea–hypopnea index, chronic kidney disease, hypertension stage, resistant hypertension, sleep apnea

## Abstract

Hypertension, blood pressure variation, and resistant hypertension have close relations to sleep apnea, which lead to target organ damage, including the kidney. The complex relationships between sleep apnea and blood pressure cause their interactions with chronic kidney disease ambiguous. The aim of the study was to elucidate the separate and joint effects of sleep apnea, hypertension, and resistant hypertension on chronic kidney disease. A cross-sectional study was done to see the associations of sleep apnea, hypertension, and resistant hypertension with chronic kidney disease in 998 subjects underwent overnight polysomnography without device-therapy or surgery for their sleep-disordered breathing. Multivariate logistic regression was used to analyze the severity of SA, hypertension stage, resistant hypertension, and their joint effects on CKD. The multivariable relative odds (95% CI) of chronic kidney disease for the aged (age ≥65 years), severe sleep apnea, stage III hypertension, and resistant hypertension were 3.96 (2.57–6.09) (*P* < 0.001), 2.28 (1.13–4.58) (*P* < 0.05), 3.55 (1.70–7.42) (*P* < 0.001), and 9.42 (4.22–21.02) (*P* < 0.001), respectively. In subgroups analysis, the multivariable relative odds ratio of chronic kidney disease was highest in patients with both resistant hypertension and severe sleep apnea [13.42 (4.74–38.03)] (*P* < 0.001). Severe sleep apnea, stage III hypertension, and resistant hypertension are independent risk factors for chronic kidney disease. Patients with both severe sleep apnea and resistant hypertension have the highest risks.

## 1 Introduction

Chronic kidney disease (CKD) is a serious global health problem,^[1]^ and patients suffering from CKD are associated with high cardiovascular morbidity and mortality.^[2]^ Recognizing and controlling the risk factors for CKD may improve outcomes.

Sleep apnea (SA) is defined as the presence of >5 complete or partial breathing disruptions, each lasting for at least 10 seconds per hour during sleep. SA is a common disease and reportedly affects ∼20% of the general population.^[3]^ Previous studies have reported a high prevalence of SA among patients with CKD, and SA has been reported to potentially be a risk factor for CKD.^[4,5]^ However, most of previous studies were conducted mainly in subjects with the estimated glomerular filtration rate (eGFR) between 60 and 90 mL/min/1.73 m^2^,^[2,6–9]^ and the effect of SA on CKD (eGFR < 60/mL/min/1.73 m^2^) is unknown. In addition, SA has been reported to be associated with hypertension and resistant hypertension,^[10,11]^ which are well-known risk factors for deteriorating renal function.^[12,13]^ SA has also been aroused to be associated with blood pressure variation^[14]^, which is closely associated with organ damage.^[15]^ Therefore, the interactions of SA, hypertension, and RH on CKD need to be clarified. The aim of the study was to clarify the interactions of SA, hypertension, and RH on CKD.

## 2 Methods

### 2.1 Patients

From January 2007 to December 2009, all patients, who underwent single night polysomnography (*Sandman* Elite System, *Tyco Inc*., *Ottawa*, *Canada*) at China Medical University Hospital, and did not undergo surgery or device-based therapy for sleep-disordered breathing, were enrolled in this study.

### 2.2 Study design

We performed this cross-sectional study to investigate the individual and synergistic effects of SA, hypertension stage, and RH on CKD. Clinical data and diagnoses were obtained from the patients’ medical history, image study, medical records, and standardized questionnaires. The data collected in from night polysomnography and medical records were reviewed by physicians, and the eGFR was calculated using the CKD–EPI formula.^[16]^ The patients with CKD were defined as those with an eGFR <60 mL/min/1.73 m^2^ for 3 months or longer.^[17]^ Patients who were ≥ 65 years were considered as “Aged.” Hypertension was defined as a blood pressure >140/90 mm Hg. Resistant hypertension (RH) was defined as a failure to adequately control blood pressure control (<140/90 mm Hg) after treatment with 3 kinds of antihypertensive drugs, of which one was a diuretic.^[18]^ The stage of hypertension was defined according to JNC7 and the Taiwan hypertension guidelines. Stage 1 hypertension was defined as systolic blood pressure/diastolic blood pressure 140–159/90–99 mm Hg, stage 2 as ≥160/ ≥100 mm Hg, and stage 3 as ≥180/ ≥110 mm Hg.^[19]^ Diabetes mellitus (DM) was defined as HbA1c>6.5% or fasting glucose ≥126 mg/dL or a random plasma glucose level ≥200 mg/dL. Hypercholesterolemia was defined as a serum cholesterol level ≥ 200 mg/dL. The body mass index (BMI) was calculated as the body weight, measured at the night of the polysomnography study, divided by the square of the height (kg/m^2^). A diagnosis of SA was according to the American Academy of Sleep Medicine 2007 manual.^[20]^ The severity of SA was estimated using the apnea–hypopnea index (AHI), with a score ≥30 been defined as severe SA, 5 to 30 as mild to moderate SA, and <5 was as no SA. A written informed consent was obtained from all the patients before commencement of polysomnography study. This study was approved by the Institutional Review Board of our hospital (reference number: DMR101-IRB2-129).

### 2.3 Statistical analysis

Descriptive statistics were presented as frequencies and percentages, or means ± standard deviations. For the comparisons of means and proportions, ANOVA (analysis of variance) tests were performed for continuous variables and trend tests were used for categorical variables. We performed multivariate logistic regression analysis, and the odds ratios (ORs) with 95% confidence intervals (CIs) were estimated after adjusting for covariates. In multivariate regression analysis, the variables were chosen based on variables that were important in the literature and clinical practice. All tests were considered statistically significant when *P* < 0.05. SAS software version 9.3 (SAS Institute, Cary, NC) was used for all data analyses.

## 3 Results

In total, 998 patients were enrolled into this study. The mean age of these participants was 51.1 ± 16.1 years and the average BMI was 26.7 ± 4.9 kg/m^2^. When the patients were classified into 3 groups according to the severity of hypertension, the mean age, BMI, and AHI score increased from the HTN (−) group (the patients without hypertension), to the group with hypertension stage 1 to 3, to the RH group. The prevalence rates of CKD and DM were significantly different among these 3 groups with the lowest rates in the HTN (–) group and highest in the RH group. There were also significant differences in gender between these 3 groups. The proportion of male was higher than the proportion of female in all the 3 groups (Table 1).

**Table 1:**
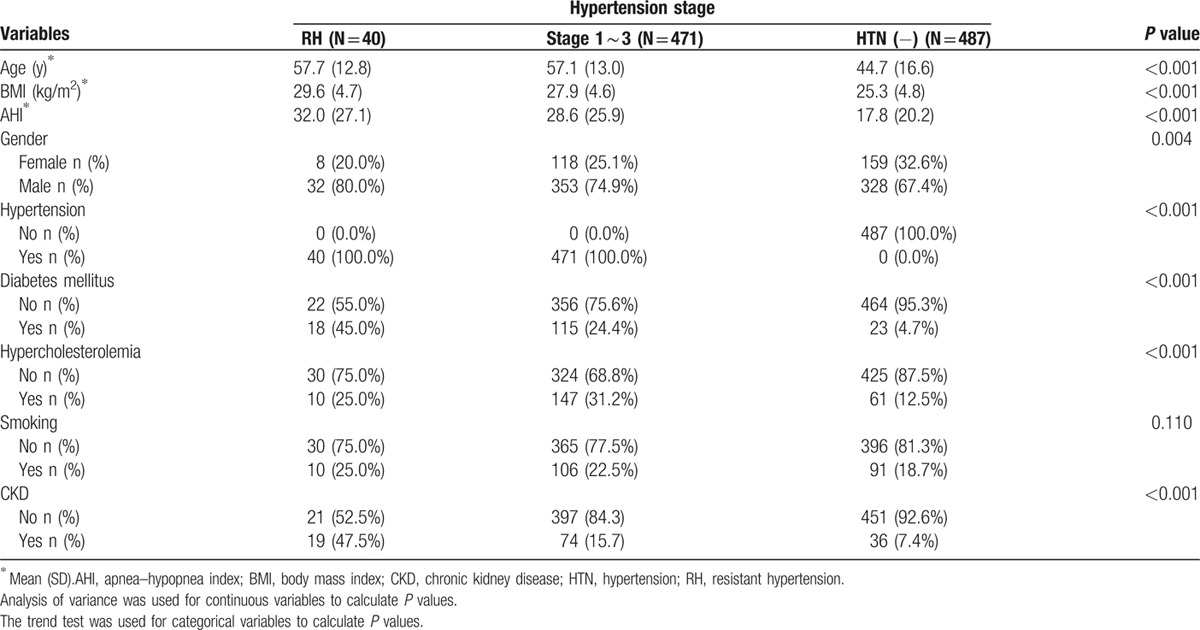
Demographic data of the 998 patients who underwent an overnight polysomography study grouped according to the severity of hypertension.

We then grouped the patients according to the severity of SA as follows: AHI ≥30 (n = 285), 5 ≤ AHI <30 (n = 499), and group with AHI<5 (n = 214). The mean age and BMI were significantly different among these 3 groups. The patients with an AHI ≥30 were older and had a higher BMI than those in the other groups. There were also significant differences in gender with more males in the AHI ≥30 group (85.6%), followed by the 5 ≤ AHI <30 group (69.7%), and AHI<5 group (56.5%). In addition, there were significant differences in hypertension, DM, and CKD between the 3 groups with higher prevalence rates in the groups. The percentages of these comorbidities were associated with groups with a higher AHI. There was no significant difference in hypercholesterolemia among the groups. There was a significant difference in smoking among the 3 groups, with the group with an AHI ≥30 having the highest percentage (24.6%) (Table 2).

**Table 2:**
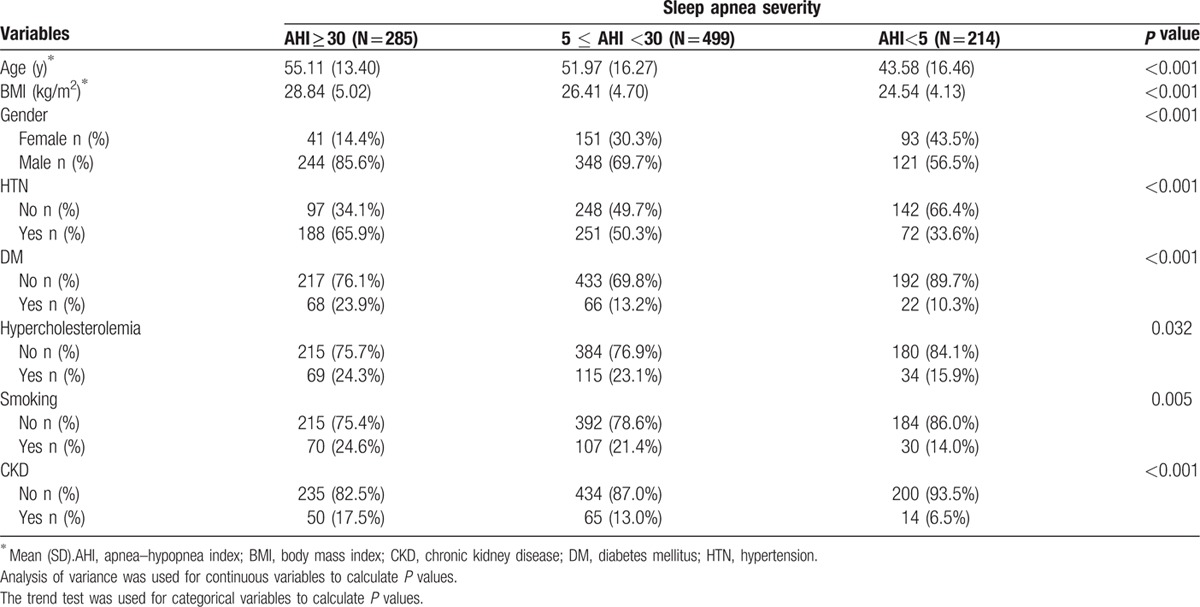
Demographic data of the 998 patients who underwent an overnight polysomngraphy grouped according to the severity of sleep apnea.

The effects of age, gender, BMI, DM, hypertension stage, RH, and severe SA on CKD were then analyzed by multivariate logistic regression. Male gender, the aged (≥65 years old), severe SA, stage 3 hypertension, and RH significantly increased the OR of CKD, whereas BMI and DM did not (Table 3).

**Table 3:**
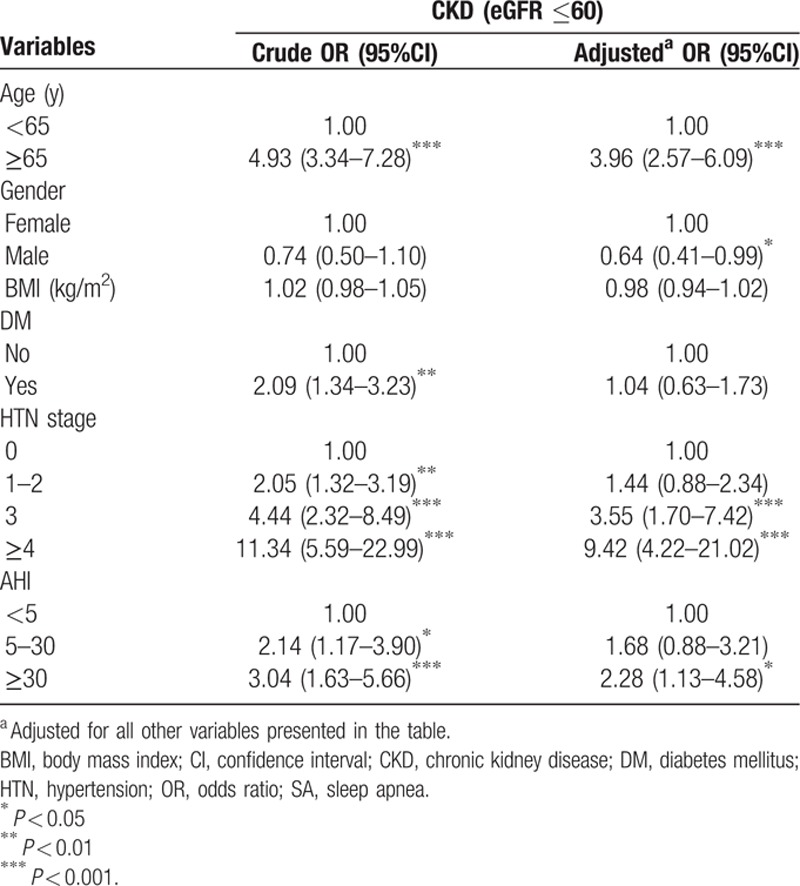
The crude and adjusted odds ratios of different variables on chronic kidney disease.

To examine the joint effects of hypertension stage, RH, and severe SA on CKD, we divided the patients into 8 subgroups according to hypertension stage and the presence of severe SA (Table 4). In multivariate analysis, the patients with RH and severe SA (RH & AHI ≥30) had the highest OR (95% CI) of CKD (13.42 [4.74–38.03]). The patients with RH without severe SA (RH & AHI <30) also had an increased OR of CKD (6.08 [2.17–16.99]). The subgroup with hypertension stage 3 with severe SA (hypertension stage 3 and AHI ≥30) had a higher OR (4.01 [1.51–10.06]) than the OR (3.09 [1.25–7.62]) of subgroup with hypertension stage 3 but without severe SA (hypertension stage 3 and AHI<30). There was only a trend of CKD in the subgroups with stage 1 and 2 hypertension. The OR of the patients with severe SA without hypertension (hypertension stage 0 and AHI ≥30) was 0.91 (95% CI = 0.39–2.10) (Table 4).

**Table 4:**
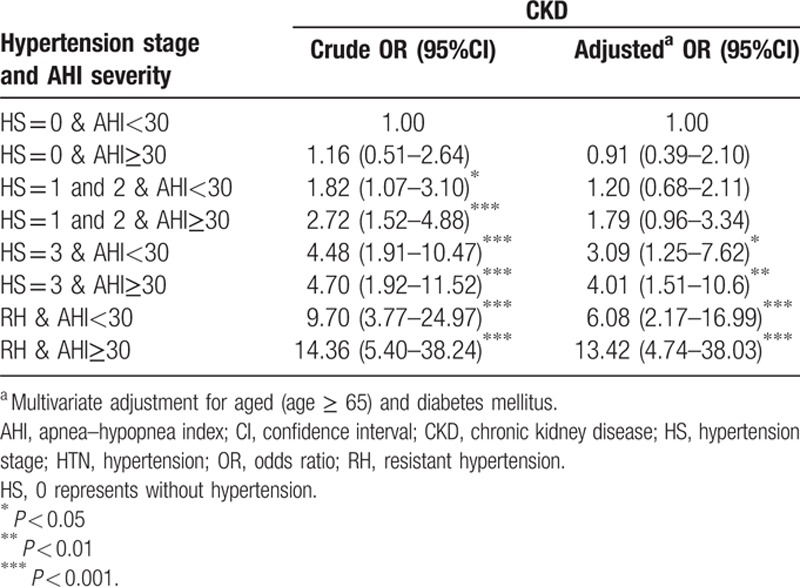
The synergistic effects of hypertension and sleep apnea on chronic kidney disease.

## 4 Discussion

To the best of our knowledge, this study is the first to report the individual and synergistic effects of SA, hypertension, and RH on CKD. Severe SA, stage 3 hypertension, RH, and the aged were the independent risk factors for CKD. In addition, the patient groups of stage 3 hypertension and RH with severe SA had a higher risk of CKD than those without it.

Age has been reported to be an independent risk factor for CKD.^[21]^ Our results also showed patients with age ≥ 65 manifested a highest OR for CKD (Table 3). Despite the significant effect of age on CKD, the individual and joint effects of severe SA, stage 3 hypertension, and RH on CKD were still found.

A previous study reported that SA may cause CKD via proteinuria and blood pressure.^[22]^ Faulx et al^[9]^ reported that proteinuria increased along with an increased AHI. In patients without hypertension and DM, those with severe SA have been reported to have a greater degree of proteinuria than those without SA. In another way, SA leads to blood pressure variation as AHI increased. Severe SA has also been reported to increase the frequency of blood pressure surge, resulting in more renal injury.^[23]^ These findings may explain why severe SA is as an independent risk factor for CKD.

Hypertension is a traditional risk factor for CKD,^[24]^ and the stage of hypertension has been positively correlated with the severity of endothelial dysfunction, arterial stiffness, and organ damage.^[25–27]^ However, RH, of which the prevalence is more than appreciated, predisposes patients to an even greater risk of organ damage.^[18]^ Consistent with those findings, we found that the patients with stage 3 hypertension and RH had a higher risk of CKD than those without hypertension. As SA was an independent risk factor for hypertension and RH,^[10,11]^ we did the research and documented the synergistic effects of severe SA, stage 3 hypertension, and RH on CKD.

We used hypertension stage and RH to analyze the effect of hypertension on CKD because blood pressure variation is common in patients with SA. The design or our study was different from previous SA studies in which only 1 or 2 blood pressure measurements were taken to assess the effect of hypertension on CKD.^[8,28]^ The same value of blood pressure in patients using 1 to 3 kinds of full-dose anti-hypertensive drugs does not mean the same risk of CKD and we think that our analysis more accurately reflects the effects of hypertension on organ damage.

Although DM is a known risk factor for CKD,^[29]^ we did not find an increased OR of DM (Table 3). Recent studies have shown that SA is not rare in patients with DM.^[30,31]^ However, previous studies of about DM on CKD have not taken the effects of SA, blood pressure variation, and RH into consideration.^[32–34]^ Further studies in DM-associated CKD should consider these factors.

## 5 Limitations

Complete data on proteinuria were lacking in this study. We did not use the data of UPCR (spot urine protein/creatinine ratio), as this is not reliable in the older patients with low muscle mass^[35]^ and it was impractical to collect 24-hour urine, the standard method to estimate proteinuria, during a polysomnography study. Further cohort studies are needed to verify our finding.

## 6 Conclusion

The present study explored the effects of SA, hypertension, and RH on CKD. The patients with stage 3 hypertension and RH had a higher risk of CKD, especially when also had severe SA. It is necessary to do polysomnography in patients with disordered sleep, and severe SA may need to be treated because of its high CKD risk.
